# Effects of Mental Fatigue in Total Running Distance and Tactical Behavior During Small-Sided Games: A Systematic Review With a Meta-Analysis in Youth and Young Adult's Soccer Players

**DOI:** 10.3389/fpsyg.2021.656445

**Published:** 2021-03-17

**Authors:** Filipe Manuel Clemente, Rodrigo Ramirez-Campillo, Daniel Castillo, Javier Raya-González, Ana Filipa Silva, José Afonso, Hugo Sarmento, Thomas Rosemann, Beat Knechtle

**Affiliations:** ^1^Escola Superior Desporto e Lazer, Instituto Politécnico de Viana Do Castelo, Rua Escola Industrial e Comercial de Nun'Álvares, Viana Do Castelo, Portugal; ^2^Instituto de Telecomunicações, Delegação da Covilhã, Lisboa, Portugal; ^3^Human Performance Laboratory, Department of Physical Activity Sciences, Universidad de Los Lagos, Santiago, Chile; ^4^Centro de Investigación en Fisiología del Ejercicio, Facultad de Ciencias, Universidad Mayor, Santiago, Chile; ^5^Faculty of Health Sciences, Universidad Isabel I, Burgos, Spain; ^6^N2i, Polytechnic Institute of Maia, Maia, Portugal; ^7^The Research Centre in Sports Sciences, Health Sciences and Human Development (CIDESD), Vila Real, Portugal; ^8^Centre for Research, Education, Innovation and Intervention in Sport, Faculty of Sport of the University of Porto, Porto, Portugal; ^9^University of Coimbra, Research Unit for Sport and Physical Activity, Faculty of Sport Sciences and Physical Education, Coimbra, Portugal; ^10^Institute of Primary Care, University of Zurich, Zurich, Switzerland; ^11^Medbase St. Gallen Am Vadianplatz, St. Gallen, Switzerland

**Keywords:** football, athletic performance, drill-based games, conditioned games, decision-making, motor skill, motor learning

## Abstract

**Background:** Mental fatigue can impact physical demands and tactical behavior in sport-related contexts. Small-sided games (SSGs) are often used to develop a specific sport-related context. However, the effects of mental fatigue on physical demands and tactical behaviors during soccer SSGs have not been aggregated for systematical assessment.

**Objective:** This systematic review (with a meta-analysis) was conducted to compare the effects of mental fatigue vs. control conditions in terms of the total running distance and tactical behavior of soccer players during SSGs.

**Methods:** The data sources utilized were PubMed, PsycINFO, Scielo, Scopus, SPORTDiscus, and Web of Science. The study eligibility criteria were established based on PICOS: (i) Population: healthy youth and young adult men soccer players with regular training practice and belonging to teams with regular competitions; (ii) Intervention: exposed to mental fatigue-induced protocols only before SSGs; (iii) Comparator: control conditions (passive or active not promoting mental fatigue) before SSGs; (iv) Outcomes: physical demands (total running distance) and tactical behavior (attacking behavior accuracy, pass decision-making accuracy, and space exploration index); (v) Study design: counterbalanced cross-over design; and (vi) only full-text and original articles written in English.

**Results:** The database search initially identified 111 titles. From those, six articles were eligible for the systematic review and meta-analysis. Results showed no significant effect of fatigue on total running distance (ES = 0.13; *p* = 0.307) and tactical behavior (ES = 0.56; *p* = 0.079).

**Conclusions:** A non-significant effect of mental fatigue on total running distance and tactical behaviors performed by soccer players during SSGs was found in this systematic review.

## Introduction

Mental fatigue is defined as a psychobiological state in which feelings of tiredness and a lack of energy occur, often after long periods of highly demanding cognitive activity or stress (Boksem et al., 2005). Due to high cognitive efforts, mental fatigue can occur caused by neuromodulation associated with adenosine (Martin et al., 2018). Considering that perception of effort and cerebral adenosine increases, while dopamine and motivation decrease, a consequence is a potential impairment in performance (Martin et al., 2018). Mental fatigue generally causes lapses in attention (impairing attentional focus) (Boksem et al., 2005), decreases cognitive performance (Russell et al., 2019), or delays one's reaction time (Jaydari Fard et al., 2019). In the sports performance context, mental fatigue seems to consistently impair endurance performance (Martin et al., 2018), while its effects on strength/power performance are inconclusive (Van Cutsem et al., 2017). In the case of team sports (e.g., soccer), it may also negatively impact technical performance (Badin et al., 2016), tactical behaviors (Kunrath et al., 2020), and decision-making abilities (Smith et al., 2016b). Considering such a list of potential effects, growing attention has been focused on the effects of mental fatigue on players' performance.

Specifically on soccer, a recent study analyzed the evidence regarding the effects of mental fatigue on the physical and tactical/technical performance of players (Smith et al., 2018). This condition likely impairs soccer-specific physical, technical, decision-making, and tactical performance (Smith et al., 2018). In terms of physical performance, mental fatigue might impair intermittent high-intensity running performance (Smith et al., 2016a). In addition, more errors occur in tactical behavior, and decisions are made more slowly and less accurately during states of mental fatigue (Smith et al., 2016b). Such evidence should be considered by coaches, especially when considering specific strategies that might help to mitigate the exposure of players to mental fatigue, as well as when adjusting training strategies that might simulate such conditions.

One of the most popular drill-based training exercises on soccer is the small-sided games (SSGs) (Clemente et al., 2021). These games are often used by coaches to develop specific tactical/technical behaviors of players while producing a physiological and physical stimulus (Sarmento et al., 2018; Clemente and Sarmento, 2020; Clemente et al., 2020a). These games simplify the complexity of official matches while maintaining the main dynamics and properties (Davids et al., 2013). If mental fatigue impairs match performance, it is likely to also impair performance in training scenarios, namely during drills designed to introduce tactical issues as the SSGs. For that reason, it is expected that mental fatigue also impairs performance during SSGs, thus possibly constraining the efficacy of the drill.

With this idea in mind, some original researches have compared the effects of mental fatigue vs. control conditions on soccer players' performance during SSGs (Badin et al., 2016; Coutinho et al., 2017; Kunrath et al., 2020). Although different outcomes have been explored during comparisons, there are some common ones (Badin et al., 2016; Coutinho et al., 2017; Kunrath et al., 2020). The most common outcomes are related to total distance (the distance covered during the SSGs) and tactical behavior (measured by using observation analysis, —namely looking for specific behavior with or without the ball—or by positional data—namely searching for exploration on the field) (Badin et al., 2016; Coutinho et al., 2017; Kunrath et al., 2020). As an example, some of the studies researching the effects of mental fatigue on SSGs revealed potential influence on collective dynamics (Coutinho et al., 2018; Kunrath et al., 2018), while apparently not in running performance (Badin et al., 2016).

Two articles have summarized the evidence of mental fatigue in soccer (Smith et al., 2018; Thompson et al., 2019). However, neither one followed a systematic review methodology considering the preferred reporting items for systematic reviews and meta-analyses (PRISMA) guidelines. Additionally, neither article (Smith et al., 2018; Thompson et al., 2019) summarized the effects of mental fatigue on the physical and tactical behavior of soccer players during SSGs.

A systematic review with a meta-analysis about the effects of mental fatigue (vs. control conditions) on physical and tactical performance could help to identify the best ways to manage such effects, thereby optimizing performance during SSGs. Therefore, the purpose of the present systematic review and meta-analysis was to assess the effects of mental fatigue on youth and young adult men soccer players in terms of total running distance and tactical performance during SSGs. In particular, within- and between-condition analyses will be executed to fulfill the main purpose of the study.

## Methods

This systematic review followed the Cochrane Collaboration guidelines (Higgins et al., 2019), and the systematic review strategy was conducted according to PRISMA guidelines (Moher et al., 2009). The PICOS (population or problem; intervention or exposure, comparison, outcome, and study design) is as follows: (P) soccer players of any age and sex who train regularly and belong to teams with regular competitions; (I) exposure to mental fatigue-induced protocols before SSGs; (C) control conditions (passive or active, not promoting mental, or physical fatigue) before SSGs; (O) physical demands (total distance as the most representative external load measure reported in the literature) and tactical behavior (attacking behavior accuracy, which is a determinant of the main goal of the game—attack to score; decision-making passes accuracy; space exploration index); and (S) a counterbalanced cross-over design. The protocol was published in (International Platform of Registered Systematic Review and Meta-analysis Protocols), with the identification number 202110014 and DOI 10.37766/inplasy2021.1.0014.

### Eligibility Criteria

Inclusion and exclusion criteria for this systematic review and meta-analysis can be found in Table 1.

**Table 1:** Inclusion and exclusion criteria (PICOS).

	**Inclusion criteria**	**Exclusion criteria**
Population	Youth or young adult (<23 years old since in some countries is the limit age for final transition to professional adult soccer) soccer players who undergo regular training practice and belong to teams with regular competitions.	Sports other than soccer; players with injuries or illnesses; women soccer; professional adult soccer.
Intervention	Exposure to mental-fatigue-inducing protocols before SSGs.	No exposure to mental-fatigue-inducing protocols before SSGs (e.g., physical fatigue before SSGs, mental fatigue tested only after SSGs). Studies monitoring mental fatigue during SSGs but not exposing players to mental fatigue conditions beforehand.
Comparator	Control conditions (passive or active but not inducive of mental fatigue) before SSGs (e.g., physical fatigue can be considered as active control).	Not compared with a passive control or an alternative protocol that is also inducive of mental or physical fatigue.
Outcome	Total running distance and tactical behavior (attacking behavior accuracy, decision-making passes accuracy; space exploration index).	No data comparing control vs. mental fatigue related to total running distance and tactical behavior.
Study design	A counterbalanced cross-over design (randomized and non-randomized can be included since neither reveals significant differences in control conditions) was considered to identify the effect of mental fatigue vs. non-fatigue in the same subjects.	Other study designs that do not allow comparisons within-subjects for the two conditions (control and mental fatigue prior SSGs).
Additional criteria	Only original and full-text studies written in English.	Not written in English. Non-original, full research articles (e.g., reviews, letters to editors, trial registrations, proposals for protocols, editorials, book chapters, and conference abstracts).

The screening of the title, abstract and reference list of each study to locate potentially relevant studies was independently performed by two authors (DC and JRG). Additionally, they reviewed the full version of the included papers in detail to identify articles that met the selection criteria. A discussion was made in the cases of discrepancies regarding the selection process.

### Information Sources

Electronic databases (PubMed, PsycINFO, Scielo, Scopus, SPORTDiscus, and Web of Science) were searched for relevant publications prior to the December 26 of 2020 (exact day of the search) and not limited to the date of publication. Keywords and synonyms were entered in various combinations in all fields: (soccer OR football) AND (“small-sided games” OR “conditioned games” OR “SSG” OR “drill-based games” OR “small-sided conditioned games” OR “reduced games” OR “play formats”) AND (“mental fatigue” OR “cognitive fatigue” OR “cognitive effort” OR “mental demands”). Additionally, the reference lists of the studies retrieved were manually searched to identify potentially eligible studies not captured by the electronic searches. Finally, an external expert was contacted to verify the final list of references included in this systematic review and to indicate if there was any study that was not detected through our research.

### Data Extraction

A data extraction was prepared in Microsoft Excel sheet (Microsoft Corporation, Readmon, WA, USA) in accordance with the Cochrane Consumers and Communication Review Group's data extraction template (Group, 2016). The Excel sheet was used to assess inclusion requirements and subsequently tested for all selected studies. The process was independently conducted by two authors (FMC and HS). Any disagreement regarding study eligibility was resolved in a discussion. Full text articles excluded, with reasons, were recorded. All the records were stored in the sheet.

### Data Items

Aiming to establish consistency in data analyzing and reporting, only measures that were analyzed three or more times for different articles were included. Two main outcomes were considered for extraction: (i) total running distance; and (ii) tactical behavior (considered as individual behavior following tactical principles or collective dynamics using data position information). For the case of the total running distance [measured in meters (absolute values), or meters per minute (standardized value)] was included since is the most valid and reliable measure (in microtechnology systems) among those measures related to time-motion analysis, while is often reported across the studies. For the case of tactical behavior, the criteria for data extraction were: (i) individual measure (related to a player); (ii) measure a behavior with ball; (iii) measure the accuracy of a behavior related with the ball.

The following information was extracted from the included original articles: (i) type of study design and randomization, number of participants (n), age-group (youth, adults, or both), sex (men, women, or both), competitive level (if available); (ii) identification of mental-fatigue induced intervention (e.g., task, time, and procedures) and control condition; (iii) identification of the SSGs (e.g., format, pitch dimensions, and rules) and training regimen implemented (work duration, work intensity, modality, relief duration, relief intensity, repetitions and series, between-set recovery).

### Assessment of Methodological Quality

The quality assessment standard for a cross-over study was used (Ding et al., 2015). This tool assesses nine items: (i) appropriate cross-over design; (ii) randomized treatment order; (iii) carry-over effect; (iv) unbiased data; (v) allocation concealment; (vi) blinding; (vii) incomplete outcome data; (viii) selective outcome reporting; and (ix) other bias. A possible scoring of low, unclear, and high can be provided by each item. Two of the authors (DC and JRG) independently screened and rated the included articles (Ding et al., 2015). The agreement between the authors was tested using the *k* agreement rate. The Cohen's kappa coefficient (*k*) executed revealed a *k* agreement of *k* = 0.92, thus suggesting an excellent reliability (Koo and Li, 2016).

### Summary Measures, Synthesis of Results, and Publication Bias

Although two studies can be used in meta-analyses (Valentine et al., 2010), considering reduced sample sizes are common in the sports science literature (Abt et al., 2020), including in SSG studies (Zouhal et al., 2020), analysis and interpretation of results in this systematic review and meta-analysis were only conducted in the case of at least three study groups provided baseline and mental fatigue-related data for the same measure. Means and standard deviation (SD) for dependent variables were used to calculate effect sizes (ES; Hedge's *g*) for each outcome in the mental-fatigue and control conditions. In case means and SDs were not available, they were obtained from 95% confidence intervals (CIs) or standard error of mean (SEM), using Cochrane's RevMan Calculator. Data were standardized using post-intervention SD values. The random-effects model was used to account for differences between studies that might impact the SSG-based effect (Deeks et al., 2008; Kontopantelis et al., 2013). The ES values are presented with 95% confidence intervals (CI). Calculated ES were interpreted using the following scale: <0.2, trivial; 0.2–0.6, small; >0.6–1.2, moderate; >1.2–2.0, large; >2.0–4.0, very large; >4.0, extremely large (Hopkins et al., 2009). Heterogeneity was assessed using the *I*^2^ statistic, with values of <25%, 25–75%, and >75% considered to represent low, moderate, and high levels of heterogeneity, respectively (Higgins and Thompson, 2002). The risk of bias was explored using the extended Egger's test (Egger et al., 1997). To adjust for publication bias, a sensitivity analysis was conducted using the trim and fill method (Duval and Tweedie, 2000), with L0 as the default estimator for the number of missing studies (Shi and Lin, 2019). All analyses were carried out using the Comprehensive Meta-Analysis software (version 2; Biostat, Englewood, NJ, USA). Statistical significance was set at *p* ≤ 0.05.

## Results

### Study Identification and Selection

**Figure 1:**
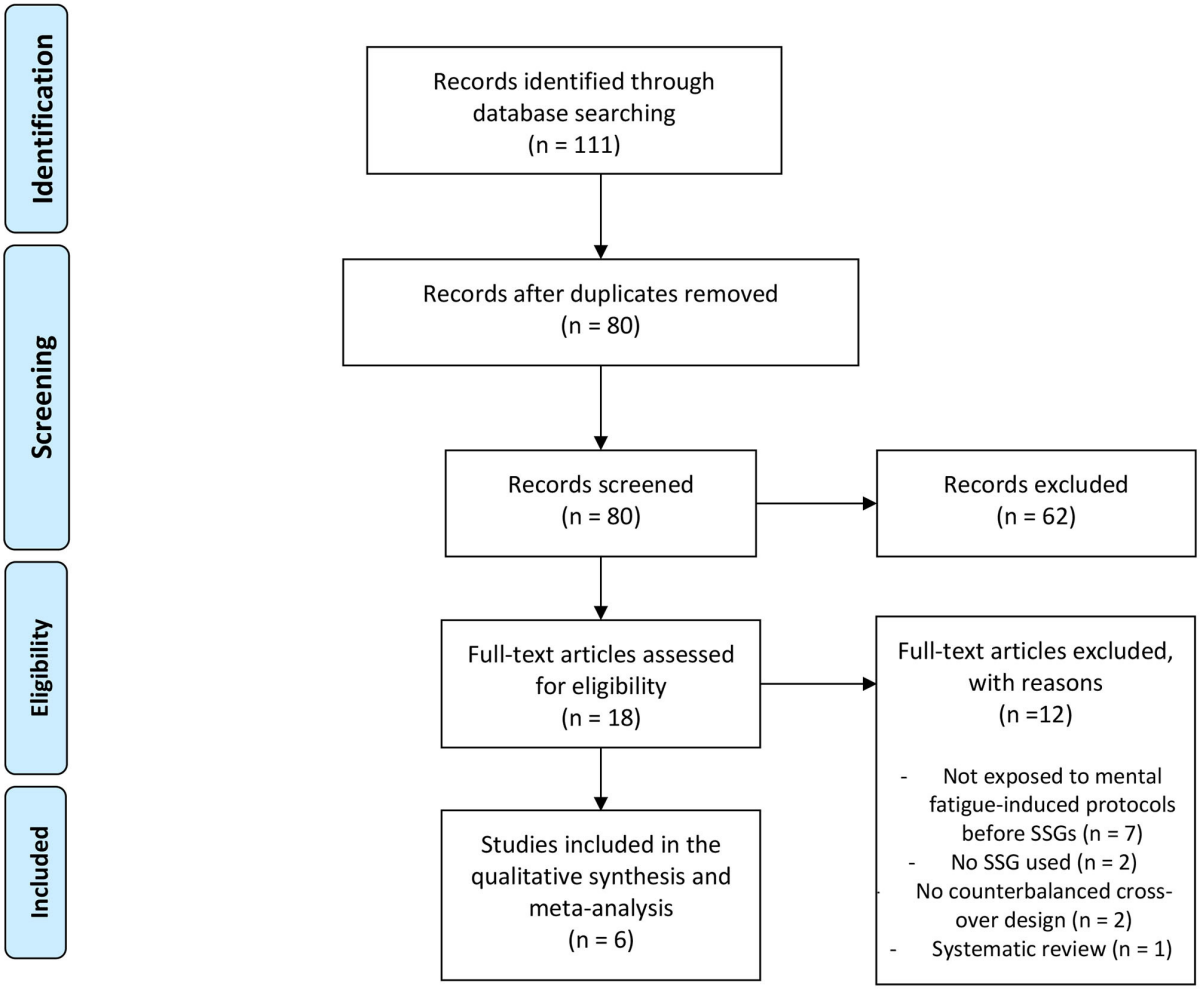
PRISMA flow diagram highlighting the selection process for the studies included in the systematic review.

The searching of databases identified a total of 111 titles. These studies were then exported to reference manager software (EndNote^TM^ X9, Clarivate Analytics, Philadelphia, PA, USA). Duplicates (31 references) were subsequently removed either automatically or manually. The remaining 80 articles were screened for their relevance based on titles and abstracts, resulting in the removal of a further 62 studies. Following the screening procedure, 18 articles were selected for in depth reading and analysis. After reading full texts, a further 12 studies were excluded due to not meet the eligibility criteria and 6 studies were selected for the further analysis (Figure 1).

### Study Characteristics

The characteristics of the included studies can be found in Table 2, while Table 3 presents the characteristics of the mental fatigue inducing protocols, control conditions and SSGs. From the included studies, only one tested the effects on adults (Kunrath et al., 2020). Five of the six studies used the Stroop task to induce mental fatigue (Badin et al., 2016; Coutinho et al., 2018; Kunrath et al., 2018, 2020; Trecroci et al., 2020). From the included studies, only two described the sex of participants (both men) (Kunrath et al., 2018, 2020).

**Table 2:** Characteristics of the included studies.

**Study**	***N***	**Sex**	**Age (y)**	**Experience (y)**	**Competitive level**	**Physical outcome extracted for this review**	**Instrument used to measure total running distance**	**Tactical outcome extracted for this review**	**Instrument used to measure tactical outcome**
Badin et al. (2016)	20	ND	17.8 ± 1.0	8.3 ± 1.4	Highest state-level Australian competition	Total distance (m)	15-Hz GPS	-	-
Coutinho et al. (2017)	12	ND	15.9 ± 0.8	8.9 ± 2.4	Amateur	Total distance (m)	15-Hz GPS	Space Exploration Index (m)	15-Hz GPS
Coutinho et al. (2018)	10	ND	13.7 ± 0.5	6.1 ± 0.9	Amateur	Total distance (m)	5-Hz GPS	Stretch Index (m)	5-Hz GPS
Kunrath et al. (2018)	6	Men	14.7 ± 0.6	-	Amateur	Total distance (m)	15-Hz GPS	Total offensive principles (%)	FUT-SAT
Kunrath et al. (2020)	18	Men	21.8 ± 2.5	-	College Athletes	Total distance (m)	15-Hz GPS	Total offensive principles (%)	FUT-SAT
Trecroci et al. (2020)	9	ND	17.6 ± 0.5	-	Semi-professional	Total distance (m)	15-Hz GPS	-	-

*ND, not defined; y, years of age; GPS, global positioning system; FUT-SAT, the system of tactical assessment in soccer (observational tool)*.

**Table 3:** Experimental approaches used among the included studies to assess the effects of mental fatigue in total running distance and tactical behavior during small-sided soccer games.

**Study**	**Instrument to induce fatigue**	**Duration of fatigue-induced protocol (min)**	**Moment of application for fatigue-induced protocol**	**Control (active and passive)**	**Format of the SSG**	**Pitch dimensions (m)**	**Rep (*n*)**	**Duration of rep (min)**	**Recov (min)**	**Observations**
Badin et al. (2016)	Stroop task	30	Immediately prior to SSGs	Documentary watching	5 vs. 5	30 × 20	2	7	1	Ball possession game
Coutinho et al. (2017)	Coordination-based task	20	Immediately prior to SSGs	Passive control	6 vs. 6 + GK	62 × 43	3	6	3	Effects of reference lines in the pitch were also compared with mental fatigue effect
Coutinho et al. (2018)	Stroop task	30	Immediately prior to SSGs	Passive control	5 vs. 5 + GK	30 × 25	3	6	3	All the conditions (control, mental fatigue and physical fatigue were made in the same session)
Kunrath et al. (2018)	Stroop task	20	40 s before the SSGs	Passive control	3 vs. 3 + GK	36 × 27	3	4	ND	Seven days of difference between control and mental fatigue conditions
Kunrath et al. (2020)	Stroop task	ND	3 min before the SSGs	Documentary watching	3 vs. 3 + GK	36 × 27	3	4	ND	Control and mental fatigue conditions were performed in two consecutive days
Trecroci et al. (2020)	Stroop task	30	10 min before the SSGs	Documentary watching	4 vs. 4 + 1	40 × 32	2	7	1	

*Rep., Repetitions; n, number; min, minutes; Recov., recovery between repetitions; GK, goalkeeper; m, meters; min, minutes; ND, not defined; SSG, small-sided games*.

### Methodological Quality

The overall methodological quality of the intervention studies can be found in Table 4.

**Table 4:** Analysis of the selected studies' methodological quality (*n* = 6).

**Study**	**1**	**2**	**3**	**4**	**5**	**6**	**7**	**8**	**9**
Badin et al. (2016)	*Unclear*	*Unclear*	*Unclear*	*Low*	*Unclear*	*High*	*Low*	*Low*	*Unclear*
Coutinho et al. (2017)	*Unclear*	*Unclear*	*Unclear*	*Low*	*Unclear*	*High*	*Low*	*Low*	*Unclear*
Coutinho et al. (2018)	*Unclear*	*Unclear*	*Unclear*	*Low*	*Unclear*	*High*	*Low*	*Low*	*Unclear*
Kunrath et al. (2018)	*Unclear*	*Unclear*	*Unclear*	*Low*	*Unclear*	*High*	*Low*	*Low*	*Unclear*
Kunrath et al. (2020)	*Unclear*	*Unclear*	*Unclear*	*Low*	*Unclear*	*High*	*Low*	*Low*	*Unclear*
Trecroci et al. (2020)	*Unclear*	*Unclear*	*Unclear*	*Low*	*Unclear*	*High*	*Low*	*Low*	*Unclear*

*(1) Appropriate cross-over design; (2) Randomized treatment order; (3) Carry-over effect; (4) Unbiased data; (5) Allocation concealment; (6) Blinding; (7) Incomplete outcome data; (8) Selective outcome reporting; and (9) Other bias*.

### Mental-Fatigue vs. Control Conditions: Effects on Total Running Distance

The synthesis of results of total running distance can be found in Table 5.

**Table 5:** Total running distance during small-sided soccer games under control (rested) and experimental (mental fatigue) conditions.

**Study**	**Control condition (mean ± SD)**	**Mental fatigue condition (mean ± SD)**	**Mental fatigue condition-Control condition (****%)**
Badin et al. (2016)	1531 ± 125	1531 ± 142	0.0
Coutinho et al. (2017)	115.0 ± 13.8	112.2 ± 13.6	−2.4
Coutinho et al. (2018)	502.1 ± 72.4	485.6 ± 90.1	−3.3
Kunrath et al. (2018)	1316.8 ± 94.9	1398.0 ± 77.6	6.2
Kunrath et al. (2020)	1335.9 ± 94.3	1375.4 ± 105.0	3.0
Trecroci et al. (2020)	949.3 ± 372.5	1050.6 ± 391.8	10.7

**Figure 2:**
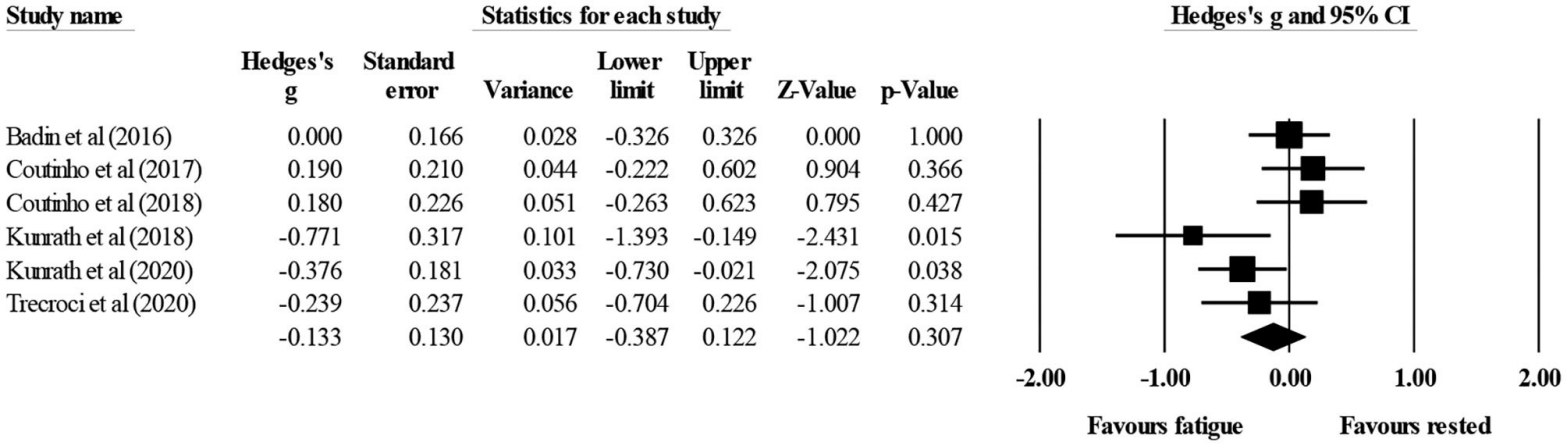
Forest plot of changes in total running distance during small-sided soccer games under control (rested) condition compared to fatigue-induced condition. Values shown are effect sizes (Hedges's g) with 95% confidence intervals (CI). The size of the plotted squares reflects the statistical weight of the study. Black diamond: overall result.

Six studies provided data for physical demands (i.e., total running distance) during small-sided soccer games, involving 6 experimental (fatigue) conditions and 6 control (rested or watching documentaries) conditions (pooled *n* = 75). There was no significant effect of fatigue on total running distance (ES = 0.13; 95% CI = −0.12 to 0.39; *p* = 0.307; *I*^2^ = 54.2%; Egger's test *p* = 0.440; Figure 2). The relative weight of each study in the analysis ranged from 10.9% to 20.8%.

### Mental-Fatigue vs. Control Conditions: Effects on Tactical Behavior

The synthesis of results for tactical behavior can be found in Table 6.

**Table 6:** Tactical behavior performance during small-sided soccer games under control (rested) and experimental (mental fatigue) conditions.

**Study**	**Control condition (mean ± SD)**	**Mental fatigue condition (mean ± SD)**	**Mental fatigue condition-control condition (****%)**
Coutinho et al. (2017)	11.8 ± 2.0	11.8 ± 2.5	0.0
Coutinho et al. (2018)	5.49 ± 0.56	5.11 ± 0.45	−6.9
Kunrath et al. (2018)	94.4 ± 2.8	93.5 ± 4.3	−1.0
Kunrath et al. (2020)	90.1 ± 5.7	66.2 ± 15.8	−26.5

**Figure 3:**
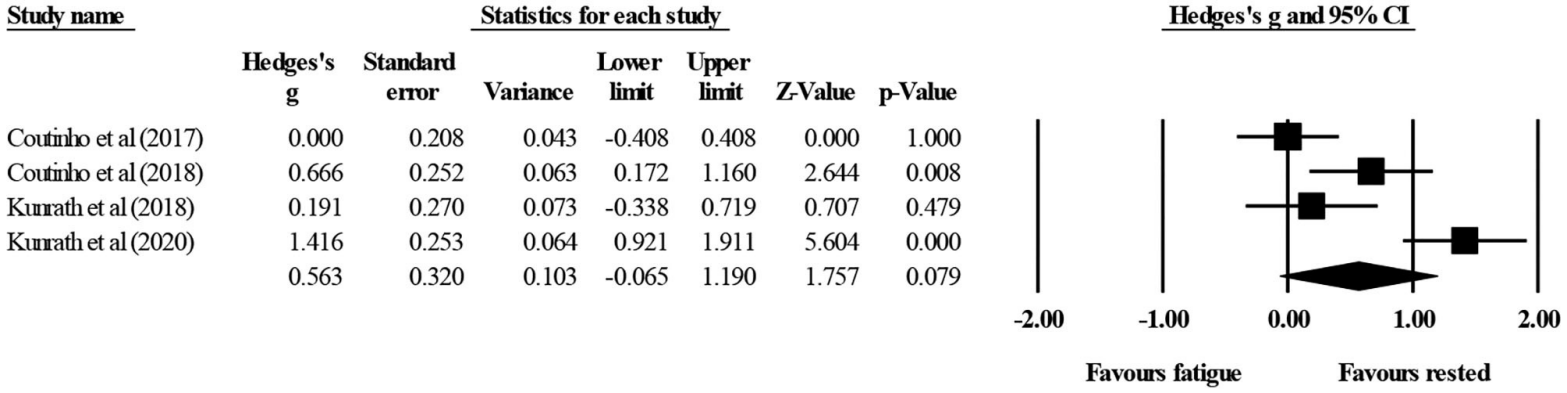
Forest plot of changes in tactical behavior during small-sided soccer games under control (rested) condition compared to fatigue-induced condition. Values shown are effect sizes (Hedges's g) with 95% confidence intervals (CI). The size of the plotted squares reflects the statistical weight of the study. Black diamond: overall result.

Four studies provided data for tactical behavior during small-sided soccer games, involving four experimental (fatigue) conditions and four control (rested) conditions (pooled *n* = 46). There was no significant effect of fatigue on tactical behavior (ES = 0.56; 95% CI = −0.07 to 1.19; *p* = 0.079; *I*^2^ = 85.5%; Egger's test *p* = 0.502; Figure 3). The relative weight of each study in the analysis ranged from 24.3 to 26.1%.

## Discussion

The present systematic review aimed to analyze the effects of mental fatigue on total distance and tactical behavior during SSGs. The main results revealed no significant differences between the conditions (mental fatigue vs. control) for either main outcome.

### Discussion of Evidence: Effects on Total Running Distance

The results of our meta-analysis showed that a mental fatigue-inducing protocol applied previously to performing SSGs had no significant effects on total running distance in comparison to the control groups. Since mental fatigue is a psychobiological state arising from prolonged periods of taxing cognitive activity (Marcora et al., 2009) (such as the tasks used in the included studies), this raises a number of questions. For example, could the mental fatigue protocols have a sub-threshold intensity and/or duration? Are the intervals between mental fatigue protocols and SSGs too long, thus washing out any potential effect? And perhaps more likely, could the ensuing SSGs be of insufficient intensity and/or duration for the effects of the pre-mental fatigue protocol to have an impact? Indeed, mental fatigue has been associated with the limits of exercise tolerance but not necessarily with performance within that tolerance window (Marcora et al., 2009).

Nevertheless, heterogeneity was high, and two studies (Kunrath et al., 2018, 2020) showed significant differences between groups, favoring the pre-fatigue group. In these two articles, the authors proposed that the increase in covered distance was a consequence of a decrease in the action quality of tactical behaviors (i.e., reduced tactical performance would generate less accurate decision-making and, consequently, the need for covering greater distances). Unfortunately, these studies had small sample sizes of six players (Kunrath et al., 2018) and 18 players (Kunrath et al., 2020). Thus, only tentative conclusions can be drawn.

More to the point, it should be considered that total distance might not be the best measure for capturing mental fatigue; it is possible that assessments of accelerations and/or peak velocities provide a more accurate picture. Recently, it has been discussed that measures such as heart rate, lactate accumulation, and neuromuscular function are largely unaffected by mental fatigue, even if endurance performance is affected (Martin et al., 2018). Consequently, the authors suggested that the impact of mental fatigue on endurance performance cannot necessarily be translated into such objective testing, although it could be captured by assessing the perception of effort.

Similarly, research using a time-to-exhaustion protocol in cycling showed that perception of effort increased following a mental fatigue protocol, while critical power and heart rate were not affected (Salam et al., 2018). Following this rationale, mental fatigue might acutely translate only to worse time-to-exhaustion protocols (Marcora et al., 2009), but SSGs are not generally designed to promote exhaustion. This raises an important concern: pre-training mental-fatigue-inducing protocols might not impair acute performance. However, the subjects perceived a greater effort, and perhaps post-training recovery needs to be longer. These possibilities should be addressed in future research.

In addition, we speculate that alternative mechanisms might mediate the relationship between mental fatigue, followed by engaging in SSGs—this topic deserves the attention of future research. One possibility is related to boredom, which is a negative emotion with potentially negative consequences and that has been shown to arise during low-autonomy tasks (van Hooft and van Hooff, 2018). In this vein, 20- to 30-min mental-fatigue-inducing protocols are likely to induce boredom. After the protocol, the players may be anxious to engage with actual physical practice. This may provide extra motivation, which would increase their engagement with the game and, consequently, total distance covered. However, data from the included studies showed that the greater distances were covered in very specific and heterogeneous zones—this should also be investigated through further research.

### Discussion of Evidence: Effects on Tactical Behavior

Since soccer players' performance, in terms of soccer competence, is influenced not only by physical and physiological demands but also by tactical behaviors, understanding the effects of mental fatigue on tactical teams' behavior during training scenarios (e.g., SSG) could be of great interest to coaches. Specifically, it could allow them to optimize the team's performance when adjusting training strategies.

The results of our meta-analysis showed that a mental fatigue-inducing protocol applied previously to performing SSGs had no significant effects on tactical behaviors in comparison with the control (rested) conditions. Although some research works included in the meta-analysis could serve as evidence that mental fatigue impairs tactical issues in soccer players, these findings should be interpreted with caution since no consensus has been reported in the literature. As such, two investigations observed no changes in tactical behaviors during SSGs under control (rested) and experimental (mental fatigue) conditions (Kunrath et al., 2018). In this sense, no changes in offensive total tactical action accuracy during a 6 vs. 6 + GK SSG (Kunrath et al., 2018) and in space exploration index during a 3 vs. 3 + GK SSG (Coutinho et al., 2017) were revealed concerning mental fatigue conditions when compared with the control conditions in youth soccer players.

Conversely, two studies reported significant effects in favor of control groups, as better tactical behaviors were observed in these groups than in participants in the fatigue-induced condition (Kunrath et al., 2020). In this sense, fatigued players (when compared to rested players) exhibited reduced values of the percentage of accuracy (−26.5%) in offensive unity during 3 vs. 3 + GK SSGs (Kunrath et al., 2020) and a decreased team stretch index (represented by the mean distances from each player to the teams' geometrical centers of gravity) of around 2% during a 6 vs. 6 + GK SSG (Coutinho et al., 2018). These heterogeneity results could be explained by the age and the number of players involved in the SSGs. As such, studies evidencing no changes in tactical behaviors during SSGs under control and mental fatigue conditions involved fewer than 10 players who were under 15 years old (Coutinho et al., 2017; Kunrath et al., 2018). Otherwise, 12 and 18 players (16 and 21 years old, respectively) were part of studies that showed changes in tactical behaviors when participants were mentally fatigued. Thus, further research is needed to understand the effects of inducing mental fatigue on tactical behaviors during SSGs in soccer.

When attempting to explain these contradictory results, there are some aspects to consider (e.g., the maturation stage and the tactical expertise across age-level groups). It is possible that collective behaviors are affected by age, maturation, and expertise level (Barnabé et al., 2016). In this sense, maturation could affect tactical efficacy via a better proficiency of peripherical perception, better identification of teammates, and better predictions of teammates' movements (Machado et al., 2019). Likewise, older and more experienced players move farther from the team's centroid (e.g., stretch index) in order to explore opportunities to create imbalances among their opponents and generate sufficient space to act (Silva et al., 2014; Clemente et al., 2020b). Also, occupying less area during gameplay is characteristic of younger players who try to reach the goal by immediately seeking depth rather than using ball possession and positional attacks (Barnabé et al., 2016).

### Study Limitations, Future Research, and Practical Applications

One of the limitations of the current systematic review and meta-analysis is that only English articles from certain databases were included. Another limitation is the reduced number of included articles despite also revealing that more original research should be conducted. An additional limitation is that only total distance was considered. Eventually, the mental fatigue may play a different effect on other external load measures as high-speed running or sprinting, despite literature suggest that the main effect of mental fatigue is commonly related to running distance and not power efforts (Van Cutsem et al., 2017). Future research should also consider identifying the described effects in both sexes (men and women).

No specific reference to fitness status and possible covariance effects of this factor on the main effects were analyzed in the included studies. This should be considered in future research. Future research could also analyze concurrent effects of expertise level, readiness, or well-being levels.

Additionally, mental-fatigue-inducing protocols should be compared with mental boosting protocols (e.g., meditation, music listening) in the future. The contrast between the two choices could potentially provide relevant information for the design of pre-warm-up protocols. Listening to music, for example, has been described as being used by ~82% of soccer players during pre-match activities (Thompson et al., 2020). Listening to neutral or self-selected motivational music while warming up has also been shown to improve short-term maximal performance in soccer players (Belkhir et al., 2019) in comparison with warming up without music. Even when perceived exertion remains unaltered, motivation for exercising and power output in a rowing task have been shown to increase after listening to preferred music vs. listening to non-preferred music or no music at all (Karow et al., 2020).

Mindfulness interventions have also been linked to decreased mental fatigue in soccer players (Zhu et al., 2020), but their effects when used pre-warm-up have not been addressed in the literature.

In practical terms, our data suggests that coaches can deliver mental fatigue-based protocols prior to soccer training sessions, without this negatively affecting running-based performance or tactical behavior in SSGs. If possible, soccer-specific protocols should be developed, to provide greater specificity. Furthermore, we speculate that pre-training video sessions with the purpose of analyzing the own team and/or the opponents is also likely not to impair performance in the subsequent training session. Alternatively, perhaps the SSGs used in these studies had insufficient duration and/or intensity, and so maybe coaches have room to provide longer and/or more intense soccer-specific stimuli to their players. Future studies should, however, verify if there are changes in distances covered at high-speeds. Finally, how pre-training mental fatigue-inducing protocols affect post-training recovery (i.e., whether, they require a longer recovery time) is currently unknown). Therefore, in congested weeks, coaches should be cautious when using these pre-training mental fatigue strategies, as it is possible that they accumulate with the regular training load and demand lengthier recovery periods.

## Conclusions

This systematic review and meta-analysis revealed a non-significant effect of mental fatigue on total running distance and tactical behaviors performed by soccer players during SSGs. Nevertheless, it is important to interpret the results with caution since other important outcomes (such as those related to internal load or technical skills) have not received extensive research. The effects of mental fatigue on different competitive levels, fitness status, or expertise levels might have a moderating effect and, thus, should be considered in future research and by coaches in daily practice.

## Data Availability Statement

The raw data supporting the conclusions of this article will be made available by the authors, without undue reservation.

## Author Contributions

FC lead the project, run the data search and methodological assessment, and wrote and revised the original manuscript. RR-C analyzed and interpreted the data, wrote the statistical report, and revised the original manuscript. AS, JA, and HS wrote and revised the original manuscript. DC and JR-G run the data search and methodological assessment, and wrote and revised the original manuscript. All authors contributed to the article and approved the submitted version.

## Conflict of Interest

The authors declare that the research was conducted in the absence of any commercial or financial relationships that could be construed as a potential conflict of interest.
